# Cerebellar white matter in musicians: Less is more?

**DOI:** 10.1002/alz.091270

**Published:** 2025-01-09

**Authors:** Aishwarya Ghosh, Palash K Malo, Sadhana Singh, Thomas Gregor Issac

**Affiliations:** ^1^ Centre for Brain Research, Indian Institute of Science, Bangalore, Karnataka India

## Abstract

**Background:**

Musical activity is known to act as a cognitive reserve and protect the brain against age‐related cognitive decline. The neural correlates of music perception and processing has been linked to the cerebellum, which is also known to be implicated in musical‐training related plasticity. These insights led us to explore the differences in cerebellar volumes between musicians and non‐musicians.

**Method:**

We selected 29 cognitively healthy older adults, from the Tata Longitudinal Study of Aging (TLSA) cohort, who were identified and divided into two groups: musicians (vocalists), who were formally trained for more than five years (n=13) and non‐musicians, who had no or less than five years of training (n=16). Brain imaging was performed using a 3 Tesla MRI system and cerebellar volumes were obtained by voxel‐based morphometry. The volumetric differences between male and female musicians were analysed using Mann‐Whitney U test. Generalized Linear Regression Model (GLM) was used to compare musicians to non‐musicians.

**Result:**

There was no significant difference between musicians and non‐musicians with respect to age (58.97±9, 57.83±5.31) and years of education (16.08±1.89, 16.06±4.20). Mann‐Whitney U test revealed a significant difference in the right cerebellar cortex (p=0.011), left cerebellar cortex (p=0.030) and left cerebellar White Matter (WM) (p=0.030) between male and female musicians with males having greater volume in each case. After adjusting for the Total Intracranial Volumes, GLM revealed musicians to have significantly less WM volumes in both left [β= ─777.86 (C.I. 95%); p=0.039] as well as right [β= ─1105.45 (C.I. 95%); p=0.011) cerebellum. Further, increase in years of involvement with music was positively correlated with right WM volume (0.197) and negative correlated with volumes of the right cerebellar cortex (─0.228), left cerebellar cortex (─0.257) and left cerebellar WM (─0.057), although not statistically significant.

**Conclusion:**

It is surprising to note lower cerebellar volumes in musicians. The concept of meta‐plasticity might be used to explain the current findings which are in line with previous studies where musicians with extensive training since an early age were found to have smaller cerebellar volumes.

